# Characterisation of Bacteriocins Produced by *Lactobacillus* spp. Isolated from the Traditional Pakistani Yoghurt and Their Antimicrobial Activity against Common Foodborne Pathogens

**DOI:** 10.1155/2020/8281623

**Published:** 2020-09-12

**Authors:** Mahreen Ul Hassan, Hina Nayab, Tayyab Ur Rehman, Mike P. Williamson, Khayam Ul Haq, Nuzhat Shafi, Farheen Shafique

**Affiliations:** ^1^Department of Microbiology, Shaheed Benazir Bhutto Women University, Peshawar 2500, Pakistan; ^2^Department of Molecular Biology and Biotechnology, University of Sheffield, Sheffield S10 2TN, UK; ^3^Institute of Basic Medical Sciences, Khyber Medical University, Peshawar 2500, Pakistan; ^4^Department of Zoology, University of Azad Jammu and Kashmir, Muzaffarabad 2500, Pakistan; ^5^Department of Biomedical Science, University of Sheffield, S10 2TN 72020 Mahreen Ul Hassan et al., UK

## Abstract

Lactic acid bacteria (LAB) are widely known for their probiotic activities for centuries. These bacteria synthesise some secretory proteinaceous toxins, bacteriocins, which help destroy similar or interrelated bacterial strains. This study was aimed at characterising bacteriocins extracted from *Lactobacillus* spp. found in yoghurt and assessing their bactericidal effect on foodborne bacteria. Twelve isolated *Lactobacillus* spp. were examined to produce bacteriocins by the organic solvent extraction method. Bacteriocins produced by two of these strains, *Lactobacillus helveticus* (BLh) and *Lactobacillus plantarum* (BLp), showed the most significant antimicrobial activity, especially against *Staphylococcus aureus* and *Acinetobacter baumannii*. Analysis of SDS-PAGE showed that *L*. *plantarum* and *L*. *helveticus* bacteriocins have a molecular weight of ~10 kDa and ~15 kDa, respectively. *L*. *plantarum* (BLp) bacteriocin was heat stable while *L*. *helveticus* (BLh) bacteriocin was heat labile. Both bacteriocins have shown activity at acidic pH. Exposure to a UV light enhances the activity of the BLh; however, it had negligible effects on the BLp. Different proteolytic enzymes confirmed the proteinaceous nature of both the bacteriocins. From this study, it was concluded that bacteriocin extracts from *L*. *helveticus* (BLh) can be considered a preferable candidate against foodborne pathogens as compared to *L*. *plantarum* (BLp). These partially purified bacteriocins should be further processed to attain purified product that could be useful for food spoilage and preservation purposes.

## 1. Introduction

Foodborne diseases (FBDs) have been a global concern. Despite the use of modern food preservation techniques, the rate of food-related illness still increases and is a substantial cause of death, especially in countries where there is a lack of proper food safety monitoring systems. About one-third of the world population is suffering from food-related diseases each year due to the consumption of contaminated or intoxicated food like canned food, meat, poultry, and fermented dairy products [1].

Consumer exposure to a nutritious and balanced diet has encouraged scientific research in the food industry to investigate and introduce natural compounds in food processing and preservation to reduce the use of chemicals to inhibit microbial growth and increase shelf life of food products. A careful and comprehensive work to combat the food-related diseases is a highly complex challenge that involves expertise in the field of science and technology for the safety, control, and management of food [2].

Yoghurt is one of the widely used dairy products that has been traditionally made and used for centuries around the globe (local name is *Dahi* in Pakistan). It is synthesised by milk fermentation with the help of bacteria. As a general belief for centuries, Dahi is used for curing diarrhoea in many countries including Pakistan. Many research studies have shown that yoghurt contains beneficial diversifying microflora, such as lactic acid bacteria (LAB) [3].

Lactic acid bacteria (LAB) are among the generally recognised as safe (GRAS) microorganisms and widely used as food and feed fermentation and preservatives added under strictly controlled conditions. Lactic acid bacteria, also known as Lactobacillales, are Gram-positive anaerobes that are nonsporulating rods or cocci and show a negative catalase test. During the fermentation of carbohydrates, they produce lactic acid as the primary end product and tolerate extremely low levels of pH. *Lactobacillus* is the largest genus used in the manufacturing of a variety of not only dairy products like milk, yoghurt, and cheese but also pickles, beer, wine, cider, chocolate, and other fermented foods. These bacteria are also used to produce animal feeds, e.g., silage [4].

Many strains of LAB isolated from yoghurt, which are involved in the fermentation of milk, also provide many other antimicrobial compounds such as hydrogen peroxide, diacetyl, fatty acids, reuterin (3-hydroxypropionaldehyde), ethanol, and bacteriocins. These bacteria confer health benefits by showing antagonistic activity against many pathogens. Yoghurt is considered a source of *Lactobacillus* bacteria that combats the pathogens causing certain digestive disorders and gut infections. Yoghurt is regarded as a healthy probiotic diet. Various strains of *Lactobacillus* bacteria have been isolated from yoghurt in the world in search of a novel strain with the highest efficacy [3, 4].

The bacteriocins have attracted many researchers and have been studied extensively. These small proteins or peptides (typically containing less than 60 amino acids) kill or inhibit the growth of some bacterial strains from similar or closely related species and usually have a narrow spectrum of bacterial growth inhibition [5, 6]. Based on their amino acid sequences, molecular weights, posttranslational modifications, and genetic characteristics, Gram-positive bacteriocins have been divided into four classes. Class I bacteriocins (also known as lantibiotics) are extensively posttranslationally modified and heat stable. They typically contain less than 28 amino acids (<5 kDa) and are linear or globular peptides containing the modified amino acids lanthionine and *β*-methyl lanthionine and dehydrated amino acids [7, 8]. Class II bacteriocins are not modified and typically contain 30-60 amino acids (<10 kDa). They have the unique properties of being heat stable, with the absence of nonlanthionine, and are positively charged. This category has further been divided into five subclasses [9]. Class III bacteriocins (also known as bacteriolysis) are large (>30 kDa) and heat labile. On exposure to high temperature (100°C), these bacteriocins usually get inactivated within 30 minutes. The genus *Lactobacillus* produces Class III bacteriocins such as helveticins J and V and lactacin B. Complex or Class IV bacteriocins contain a protein with lipid and carbohydrate molecules, which help in antimicrobial activity [10].

Bacteriocin-producing strains can be used as probiotics. Bacteriocin can be used as food additives, in the treatment of pathogen-associated diseases and cancer therapy [11, 12]. Indeed, certain infectious diseases caused by pathogenic strains of Gram-positive bacteria (staphylococci, micrococci, and streptococci) can be prevented by bacteriocins [13]. Moreover, many Gram-negative bacteria such as *E*. *coli*, *Salmonella*, *Shigella*, *Listeria*, and *Vibrio* have also been used as a test organism to investigate the antagonistic activity of newly isolated antimicrobial peptides. Although bacteriocin has been isolated from many strains of *Lactobacillus plantarum*, *Lactobacillus bulgaricus*, *Lactobacillus acidophilus*, and *Lactobacillus lactis*, it has shown a significant activity against a pathogen. Nevertheless, scientists are exploring novel strains from different food products to resolve the issue of most common food-related infections [12].

Increased consumer demand for natural preservatives has also made scientists focus on finding new natural inhibitors. Bacteriocin-producing LAB was used in many starter cultures to prevent pathogenic microbe from colonising many food products. These promising characteristics of bacteriocin and bacteriocin-producing LAB made them important not only for food preservation but also for treating certain drug-resistant pathogens [13, 14].

The purpose of the current study was to achieve partial purification of bacteriocins synthesised by *Lactobacillus* spp. found in locally made yoghurt samples. These partially purified bacteriocins were further compared for their physical and biochemical properties, and their antagonistic activity was tested against foodborne bacterial pathogens. The two isolates chosen for assessing bacteriocin production and activity were *L*. *helveticus* and *L*. *plantarum*.

### 1.1. Contributions

Our contributions in this study are as follows:

- In contrast to the aforementioned studies, we did partial purification, characterisation, and antibacterial activity of bacteriocin isolated from *Lactobacillus* species found in traditional yoghurt. The *Lactobacillus* species isolated from yoghurt samples were further assessed, and characterisation has shown that *L*. *helveticus* and *L*. *plantarum* strains were involved in the production of bacteriocins. The comparative study analysis on both bacteriocins showed that BLh bacteriocin was the most suitable biocontrol agent against foodborne pathogens.
- Among these two strains, bacteriocin extracted from *L*. *helveticus* was seldom found anywhere in the world. It was the first time that this strain has been isolated from a yoghurt sample, while *L*. *plantarum* has been isolated many times from dairy products. Bacteriocin from theses isolates could further be subjected to some in vivo approaches to find the actual target of the extracted bacteriocin.

## 2. Methodology

### 2.1. Bacterial Strains, Media, and Cultivation Conditions

Ten clinically identified strains (indicator organism) used in this study were ordered from the Bacteriology Lab, Department of Microbiology, at the Faculty of Sciences, Punjab University, Pakistan. The indicator strains included in this study are as follows: *Enterococcus faecium* DO, *Bacillus subtilis*, *Streptococcus pyogenes*, *Staphylococcus aureus*, methicillin-resistant *Staphylococcus aureus* (MRSA), *Escherichia coli*, *Klebsiella pneumoniae*, *Pseudomonas aeruginosa*, *Acinetobacter baumannii*, and *Salmonella paratyphi* A.

### 2.2. Isolation, Screening, and Identification of Bacteriocin-Synthesising Strains

Overall, 50 “yoghurt” samples were purchased from the local shops in loose from several urban areas in Khyber Pakhtunkhwa province of Pakistan, over three months (from January 2019 to April 2019). These traditional yoghurts were made from previously homemade yoghurts and then homogenised aseptically. Ten grams of each yoghurt sample was mixed in 50 ml of 0.9% *w*/*v* of a sterile normal saline and vortexed. Then, this mixture was serially diluted eight times. The diluted samples were plated (0.1 ml suspension) on De Man, Rogosa, and Sharpe (MRS) agar (Merck, Darmstadt, Germany) by the spread plate method. The MRS agar plates were incubated anaerobically at 37°C for a maximum of 48 hours using the BBL GasPak system. About twelve different colonies appeared on each plate, which was then subcultured on the MRS medium for further screening of bacteriocin-producing isolates.

All isolates were identified by the method described by Schillinger and Lücke [15]. API 50 CHL test strips (bioMérieux, Marcy l'Etoile, France) were used in sugar fermentation tests.

### 2.3. Detection of Antagonistic Activity

The bacteriocin-producing isolates (test organisms) were screened by the agar well diffusion method [16]. An inoculum of test organisms was added into MRS broth (50 ml) which was then subjected to 24 hrs incubation at 37°C in a shaking incubator. Following incubation, broth containing the test organisms was centrifuged at 2500 x g for 5 minutes. Following incubation, the indicator organism (MRSA bacteria) lawn was produced on an MRS agar plate by the spread plate method. Clear wells containing a diameter of 6 mm were made in the agar with the help of a sterile Pasteur pipette. An aliquot of the supernatant (~60 *μ*l) was loaded into each well, and these were labelled accordingly. The plates were then incubated at 37°C for 18-24 hrs. The plates were examined for clear zones surrounding the wells, which indicate the bacteriocin activity of the test organisms. The size of the zone of inhibition was measured in millimeters and was duly logged.

### 2.4. Extraction of Bacteriocins

The solvent extraction method of Westley et al. [17] was employed for the extraction of bacteriocins from the selected strains. To obtain a cell-free extract, the isolated bacteria were inoculated into MRS broth (100 ml) and incubated at 37°C for 24 hours. In a 500 ml separating funnel, culture broth (~50 ml) and an equal volume of ethyl acetate were added. The separating funnel was shaken vigorously for 10 minutes, and then, the content was allowed to settle, making two distinct layers. The upper organic layer that contained the bacteriocin was separated carefully. The same procedure was repeated with the rest of the broth culture, and the upper solvent layer was separated. The solvent was then removed under vacuum using a rotary evaporator. The final dried extract was dissolved in 1 ml of methanol, and the supernatant was adjusted to pH 6 with 1 M NaOH to eliminate inhibitory activity from acid and then treated with 5 mg/ml catalase to remove the antagonistic activity from hydrogen peroxide. The extract was passed through a 0.20 *μ*m pore size membrane filter (Sartorius Stedim). The filtered supernatant was stored in glass vials.

### 2.5. Activity of Extracted Bacteriocins against Indicator Strains

The antibacterial activity of extracted bacteriocin was performed by agar well diffusion assay—activity against ten indicator strains. Antibacterial activity was measured by the zone of inhibition.

### 2.6. Physical and Biochemical Characteristics of Bacteriocins

The factors affecting the antimicrobial activity of bacteriocins partially extracted from *L*. *helveticus* (BLh) and *L*. *plantarum* (BLp) were as follows.

#### 2.6.1. Temperature

All samples of bacteriocin extracts were exposed to different temperatures for 15 minutes. Then, their activities were tested using the agar well diffusion method against all indicator organisms, and activities were compared to nonexposed bacteriocins as a control.

#### 2.6.2. pH

To test how pH affects the bacteriocin activity, an aliquot of bacteriocin extract (0.5 ml) was added into MRS broth (4.5 ml) at different pH values (3 to 11) and incubated for 30 minutes at 37°C. Bacteriocin samples were exposed to different pH values and were assayed against indicator organisms by the agar well diffusion method, and activities were compared to nonexposed bacteriocins as a control.

#### 2.6.3. Bile Salts

The bile salts affecting bacteriocin activity were tested by adding an aliquot of bacteriocin extract (0.5 ml) into MRS broth (4.5 ml) at different bile salt concentrations (0.1 to 0.6%) and incubated for 30 minutes at 37°C. Bacteriocin samples exposed to different bile salt concentrations were assayed against indicator organisms by the agar well diffusion method, and activities were compared to nonexposed bacteriocins as a control.

#### 2.6.4. UV Light

Bacteriocin extracts (10 ml) were exposed to UV light of different wavelengths for the periods of 15, 30, 45, 60, and 75 minutes. Bacteriocin samples exposed to different UV light conditions were assayed against indicator organisms by the agar well diffusion method, and activities were compared to nonexposed bacteriocins as a control.

#### 2.6.5. Enzymes

The extracted bacteriocin was tested with different enzymes (1 mg/ml) and incubated for 2 h at 37°C: proteinase K, chymotrypsin, trypsin, pepsin, pronase, papain, and *α*-amylase (Sigma, USA). The control without enzyme was also incubated. The agar well diffusion method was applied to measure the antimicrobial activity.

#### 2.6.6. Size Estimation of Extracted Bacteriocins

The molecular weights of bacteriocins were estimated by 15% sodium dodecyl sulphate-polyacrylamide gel electrophoresis (SDS-PAGE) using LKB Bromma 2050 Midget electrophoresis units (Pharmacia Amersham Co.). Following electrophoresis, gels were stained with Coomassie Brilliant Blue R-250. A protein ladder (170-10 kDa) was used for estimating the molecular weights of bacteriocins.

### 2.7. Statistical Analysis

Statistical tools like the Pearson's correlation and ANOVA were applied to analyse the data. R program version 1.3.959, GraphPad Prism version 7.04, and MS Excel 2016 were used to analyse data.

## 3. Results and Discussion

### 3.1. Isolation, Screening, and Identification of *Lactobacillus* spp. from Yoghurt

Around 50 samples of yoghurt were collected from different areas of KPK, Pakistan, out of which twelve *Lactobacillus* strains were isolated and screened for antimicrobial activity against the indicator organisms. Among these 12 strains, *L*. *helveticus* and *L*. *plantarum* showed the highest antibacterial activity against MRSA. Both the strains of *Lactobacillus* species (*L*. *helveticus* and *L*. *plantarum*) were identified by the first Culture Bank of Pakistan. During the morphological characterisation, *L*. *helveticus* produced white colonies and *L*. *plantarum* produced yellow colonies on MRS agar plates. Gram staining confirmed that the *Lactobacillus* spp. were Gram-positive rods which are nonmotile and noncapsulated, with rounded ends, and did not form spores. *L*. *helveticus* showed a negative pigmentation test, while *L*. *plantarum* was positive for the test. Biochemical features of the *L*. *helveticus* and *L*. *plantarum* isolates included positive results for the citrate utilisation test and negative results for the indole, methyl red, hydrogen sulphide, and oxidase tests as shown in Table S1. The catalase test was negative for *L*. *helveticus* and positive for *L*. *plantarum*. The salt tolerance test revealed *L*. *helveticus* as NaCl tolerant, while *L*. *plantarum* failed to show the tolerance.

### 3.2. Screening for Antagonistic Activity against Indicator Organism

Bacteriocin production occurred when *Lactobacillus* spp. were in the log phase of growth. Among twelve supernatants, only two isolates showed antagonistic activity against the indicator. The agar well diffusion method revealed that *L*. *helveticus* (BLh) showed the most significant activity against MRSA after 18 to 24 hours of incubation at 37°C. In comparison, *L*. *plantarum* (BLp) showed similar activity against MRSA after 24 to 48 hours of incubation at 37°C under anaerobic conditions, as shown in Figure S1.

### 3.3. Antimicrobial Activity of Bacteriocin Extracts

**Figure 1:**
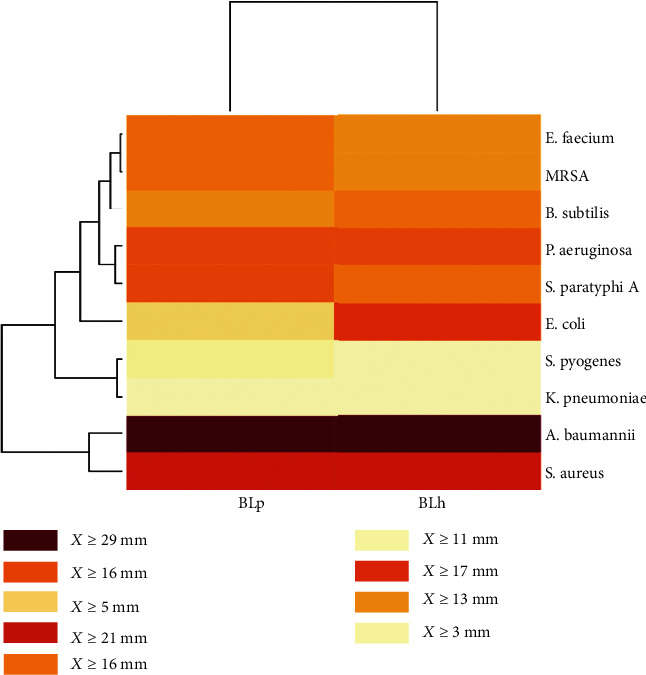
Graphical representation of the antagonistic activity map of partially purified bacteriocins against ten indicator strains. BLh stands for the bacteriocin isolated from *L*. *helveticus*, and BLp stands for bacteriocin isolated from *L*. *plantarum*.

The partial purification of bacteriocins from *Lactobacillus* sp. isolates was achieved using the organic solvent extraction method. Here, the upper ethyl acetate layer contains bacteriocins, and the lower aqueous layer contains waste material. Bacteriocin purification was not achievable through the ammonium sulphate method as no pellet was obtained during this method. The antimicrobial activities of bacteriocin extracts from *L*. *helveticus* (BLh) and *L*. *plantarum* (BLp) were tested against Gram-positive and Gram-negative indicator organisms using the agar well diffusion method, and they both showed the most significant activity against *S*. *aureus* (21.2 ± 0.1 and 19.3 ± 0.1 mm, respectively) (Figure 1 and Table S2). There was lower activity against *S*. *pyogenes* (2.6 ± 0.1 and 3.5 ± 0.1 mm) as shown in Table S3. A similar pattern for the inhibitory zone of bacteriocins against food spoilage bacteria was reported by Abo-Amer [18]. Here, *L*. *plantarum* released bacteriocin between the late log and stationary growth phases with concomitant antimicrobial activity. Consistent with our study, Abo-Amer [18] isolated *L*. *plantarum* AA135 from Egyptian homemade yoghurt, and this showed the most significant activity against foodborne Gram-positive pathogens (*S*. *epidermidis*, *S*. *aureus*, and *Bacillus cereus*) and weaker activity against *Listeria monocytogenes* and *Bacillus subtilis*. The partially purified bacteriocin isolated from *L*. *plantarum* and *Lactobacillus helveticus* showed strong activity against Gram-negative bacteria such as *E*. *coli*, *A*. *baumannii*, *P*. *aeruginosa*, and *S*. *paratyphi* A. Triplicate studies were performed on the zone of inhibition to measure the standard deviation.

### 3.4. Effects of Environmental Conditions on the Activity of *Lactobacillus* sp. Bacteriocin Extracts

The effects of temperature, pH, bile salts, and UV light on the stability and activity of bacteriocins extracted from *L*. *helveticus* (BLh) and *L*. *plantarum* (BLp) were observed. These conditions affected the antagonistic activity of bacteriocins against the Gram-positive and Gram-negative indicator organisms, which are all foodborne pathogens. Some of the conditions enhanced bacteriocin activity, while other conditions decreased their antimicrobial activity.

#### 3.4.1. Temperature

Temperature plays a crucial role in bacteriocin activity, and this was shown regarding the graph between the zone of inhibition of the indicator organisms and temperature (Tables 1–4). There was a general trend showing a decrease in bacteriocin activity with an increase in temperature with a clear difference between the two *Lactobacillus* species. The bacteriocin extracted from *L*. *plantarum* (BLp) was quite active even passing through high temperature and pressure during sterilisation, indicating it is a heat-stable protein. Abo-Amer and Fatima and Mebrouk have reported similar observations in different studies [18, 19], emphasising the usefulness of *L*. *plantarum* bacteriocin in food preservation procedures due to its high-temperature tolerance. The bacteriocin synthesised by *L*. *helveticus* (BLh) was more heat labile, withstanding temperatures of up to only 50°C for 15 minutes. Exposure to temperatures of 60°C or above for 15 minutes resulted in the loss of activity. Bacteriocins produced by both *L*. *helveticus* and *L*. *plantarum* showed the most significant activity following exposure to 30°C for 15 minutes with a broad range of activity against *S*. *aureus*, MRSA, *E*. *coli*, *S*. *paratyphi*, and *A*. *baumannii* as shown in Figures S2 and S3.

#### 3.4.2. pH

Bacteriocins produced by the isolated *Lactobacillus* spp. were active over a wide-ranging pH, with maximum activity recorded at pH 5 (Tables 5–8). Both retained antimicrobial activity following exposure to acidic conditions at pH 3 to 6 and pH 7. Following exposure to alkaline conditions at pH 8-11, the *L*. *plantarum* (BLp) bacteriocin showed a sharp decline at alkaline pH, while the *L*. *helveticus* (BLh) bacteriocin did not show any activity beyond pH 8. Similar observations were made by Sankar et al. [20], where bacteriocins maintained only partial antagonistic activity when the pH was changed from acidic to basic. Fatima and Mebrouk [19] also observed that bacteriocin synthesised by *L*. *plantarum* had maximum activity at acidic pH, and Joerger and Klaenhammer [21] reported that bacteriocin produced by *L*. *helveticus* had maximum activity at acidic pH. Bacteriocins produced by both *Lactobacillus* spp. showed the most significant activity against MRSA, *S*. *aureus*, *S*. *paratyphi*, and *E*. *coli* than to compared to the rest of the tested organisms, as shown in Figures S4 and S5. The weaker antagonistic activity was observed for *K*. *pneumoniae* and *P*. *aeruginosa* (Tables 5 and 7).

#### 3.4.3. Bile Salts

The tolerance to bile salts was assessed by exposing the bacteriocin extracts to different concentrations of bile salts (0.1 to 0.6%) (Tables 9–12). The *L*. *plantarum* (BLp) bacteriocin had a bile salt tolerance of 0.4%, while the *L*. *helveticus* (BLh) bacteriocin did not show any tolerance to 0.4% of bile salts. The *L*. *helveticus* bacteriocin was found to be highly active against Gram-positive and Gram-negative bacteria in the presence of 0.1 and 0.2% bile salts, while the bacteriocin synthesised by *L*. *plantarum* was found significantly active against indicator strains in the presence of 0.1 and 0.3% bile salts as shown in Figures S6 and S7. Previous studies have reported that *Lactobacillus* spp. showed tolerance to 0.3% bile salts, especially those that release thermally stable bacteriocins [22]. In agreement with this, the heat-labile *L*. *helveticus* bacteriocin had a bile salt tolerance of up to only 0.1%. Bile salt tolerance allows such bacteria to survive, grow, and perform useful functions in the gastrointestinal tract [23].

#### 3.4.4. UV Light

Exposure to UV light could change the nature, structure, and function of a protein. It was noticed that the bacteriocin synthesised by *L*. *helveticus* (BLh) had increased activity following UV light exposure for 30 and 15 minutes, while the bacteriocin synthesised by *L*. *plantarum* (BLp) was not affected under the same conditions as shown in Figure S8. Similar observations were described by Fatima and Mebrouk [19] and Ogunbanwo et al. [24]. The bacteriocin synthesised by *L*. *helveticus* had the highest antagonistic activity against *Acinetobacter baumannii*, *E*. *coli*, *S*. *aureus*, and MRSA following 30 minutes of UV exposure and weaker antagonistic activity against *S*. *pyogenes*, *S*. *paratyphi*, and *P*. *aeruginosa* (Tables 13 and 14). Similar results were reported by Joerger and Klaenhammer [21] on bacteriocin produced by *L*. *helveticus*.

#### 3.4.5. Enzymes

To confirm the protein nature of bacteriocins from two species, the extracts were exposed to proteolytic enzymes. The antimicrobial activity of both crude bacteriocins was completely lost after the treatment with proteinase K, chymotrypsin, trypsin, papain, pronase, and pepsin, which confirmed the proteinaceous nature. The crude extracts of both bacteriocins when treated with *α*-amylase showed no change in antimicrobial activity, indicating that carbohydrate moieties were not required for antimicrobial activity, as shown in Table 15.

### 3.5. Molecular Weights and Classification of Bacteriocins Extracted from *Lactobacillus* spp.

**Figure 2:**
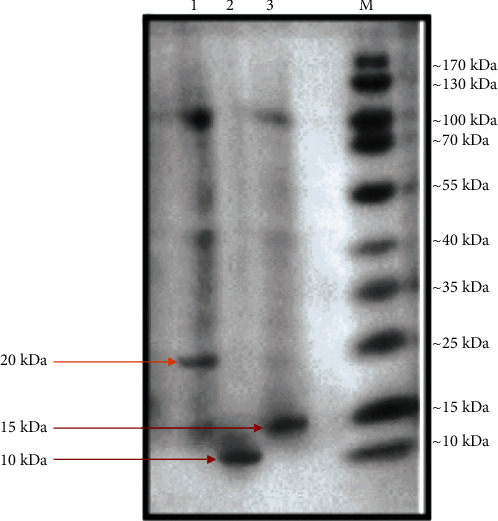
Molecular weight determination of bacteriocin by SDS-PAGE. Lane 1 contains the positive control, Lane 2 contains bacteriocin from *Lactobacillus plantarum*, Lane 3 contains bacteriocin from *Lactobacillus helveticus*, and Lane M contains the molecular weight marker.

The molecular size of bacteriocins extracted from *Lactobacillus* spp. was estimated by SDS-PAGE alongside molecular weight marker proteins (Figure 2). The bacteriocin from *L*. *plantarum* (BLp) had a molecular weight of approximately 10 kDa. Because this bacteriocin showed thermal stability, it might belong to the Class II bacteriocin family. The same molecular weight for bacteriocin synthesised by an *L*. *plantarum* strain was reported by Todorov et al. [25]. The bacteriocin from *L*. *helveticus* (BLh) had a molecular weight of approximately 15 kDa. Because this bacteriocin was thermally labile, it could belong to the Class III bacteriocin family. The same molecular weight for bacteriocin produced by an *L*. *helveticus* strain was reported by Bonadè et al. [26]. Further confirmation for the classification of bacteriocins extracted from *L*. *helveticus* and *L*. *plantarum* could be achieved by performing mass spectrometry and structural analysis of the purified proteins by NMR.

## 4. Conclusion

Entire analyses were completed in triplicate, following data compilation using R program 1.3.959, MS Excel 2016, and GraphPad Prism version 7.04. Pearson's correlation with a *P* value 0.05 was applied to the data. The data has strongly supported the alternative hypothesis, which means there was certainly some effect of bacteriocin on the indicator pathogens, especially where it showed significant values for the activity of bacteriocins during temperature variations.

In summary, bacteriocins extracted from *Lactobacillus* spp. had vigorous antimicrobial activity against Gram-positive and Gram-negative pathogenic organisms, including *S*. *aureus*, MRSA, *E*. *coli*, *Salmonella paratyphi*, and *Acinetobacter baumannii*. Various levels of activity were retained or enhanced following exposure to a range of conditions of temperature, pH, bile salts, and UV light. Based on its estimated molecular weight and the observed property of thermal stability, the bacteriocin produced by *L*. *helveticus* likely belongs to the Class II bacteriocin family. The larger and thermally labile bacteriocin produced by *L*. *helveticus* may belong to the Class III bacteriocin family. It is also concluded from our study that bacteriocin produced by *L*. *helveticus* was more effective against foodborne pathogens as compared with bacteriocin produced by *L*. *plantarum*. Further research to study the properties, structure, and mode of action of such bacteriocins is required, especially for assessing their potential use in the biocontrol of bacterial diseases and as a substitute to antibiotics.

## Figures and Tables

**Table 1:** Effect of temperature on *L*. *helveticus* (BLh) bacteriocin activity against Gram-positive bacteria.

	Gram-positive bacterial strains	Zone of inhibition (mm) vs. temperature variations (°C)									Pearson's correlation b/w temperature variations and activity (*P* value = 0.05 (two-tailed))	*R* squared (*r*^2^)
		30°C	40°C	50°C	60°C	70°C	80°C	90°C	100°C	121°C		
BLh bacteriocin	*E*. *faecium*	16.8	14.7	12.6	0.9	0.5	0.3	0.2	0.1	0.0	0.006 (significant)	0.68
	*B*. *subtilis*	18.1	13.76	12.0	0.7	0.4	0.2	0.1	0.0	0.0	0.006 (significant)	0.67
	*S*. *pyogenes*	16.4	12.80	10.8	0.6	0.3	0.1	0.0	0.0	0.0	0.006 (significant)	0.67
	MRSA	20.5	18.83	16.5	0.4	0.2	0.0	0.0	0.0	0.0	0.007 (significant)	0.65
	*S*. *aureus*	20.2	17.8	16.0	0.0	0.0	0.0	0.0	0.0	0.0	0.008 (significant)	0.65

**Table 2:** Effect of temperature on *L*. *helveticus* (BLh) bacteriocin activity against Gram-negative bacteria.

	Gram-negative bacterial strains	Zone of inhibition (mm) vs. temperature variations (°C)									Pearson's correlation b/w temperature variations and activity (*P* value = 0.05 (two-tailed))	*R* squared (*r*^2^)
		30°C	40°C	50°C	60°C	70°C	80°C	90°C	100°C	121°C		
BLh bacteriocin	*K*. *pneumoniae*	10.8	10.3	8.9	0.3	0.2	0.1	0.0	0.0	0.0	0.009 (significant)	0.64
	*P*. *aeruginosa*	12.1	11.2	10.7	0.4	0.1	0.0	0.0	0.0	0.0	0.007 (significant)	0.65
	*A*. *baumannii*	12.7	12.3	11.6	0.8	0.5	0.0	0.0	0.0	0.0	0.006 (significant)	0.67
	*S*. *paratyphi*	20.0	19.6	18.0	0.6	0.4	0.2	0.1	0.0	0.0	0.008 (significant)	0.65
	*E*. *coli*	30.2	28.8	27.4	0.9	0.3	0.1	0.0	0.0	0.0	0.008 (significant)	0.65

**Table 3:** Effect of temperature on the activity of the purified *L*. *plantarum* (BLp) bacteriocin against Gram-positive bacteria.

	Gram-positive bacterial strains	Zone of inhibition (mm) vs. temperature variations (°C)									Pearson's correlation b/w temperature variations and activity (*P* value = 0.05 (two-tailed))	*R* squared (*r*^2^)
		30°C	40°C	50°C	60°C	70°C	80°C	90°C	100°C	121°C		
BLp bacteriocin	*E*. *faecium*	16.3	14.2	13.0	11.2	10.5	8.7	6.5	4.7	2.6	*P* < 0.00001 (significant)	0.9914
	*S*. *pyogenes*	17.4	13.6	11.6	10.5	7.5	5.7	4.2	3.7	1.7	*P* < 0.00001 (significant)	0.9487
	*B*. *subtilis*	16.3	12.5	10.8	8.7	6.5	4.5	3.3	2.6	1.4	*P* < 0.000026 (significant)	0.9326
	MRSA	20.6	18.8	16.4	16.7	14.5	12.7	10.4	8.4	4.3	*P* < 0.00001 (significant)	0.9843
	*S*. *aureus*	19.7	17.7	15.4	15.7	13.5	11.6	9.5	7.4	3.3	*P* < 0.00001 (significant)	0.9841

**Table 4:** Effect of temperature on the activity of the purified *L*. *plantarum* bacteriocin (BLp) against Gram-negative bacteria.

	Gram-negative bacterial strains	Zone of inhibition (mm) vs. temperature variations (°C)									Pearson's correlation b/w temperature variations and activity (*P* value = 0.05 (two-tailed))	*R* squared (*r*^2^)
		30°C	40°C	50°C	60°C	70°C	80°C	90°C	100°C	121°C		
BLp bacteriocin	*K*. *pneumoniae*	10.8	10.3	8.9	7.7	7.03	5.8	5.4	2.3	0.0	*P* < 0.00001 (significant)	0.98
	*P*. *aeruginosa*	12.1	11.2	10.7	9.0	7.8	6.6	5.8	3.8	0.0	*P* < 0.00001 (significant)	0.97
	*E*. *coli*	30.2	28.8	27.4	26.0	24.9	23.7	22.8	10.9	0.0	*P* < 0.001 (significant)	0.77
	*A*. *baumannii*	12.7	12.3	11.6	10.56	9.0	8.0	7.1	5.66	0.0	*P* < 0.00001 (significant)	0.94
	*S*. *paratyphi*	20.0	19.6	18.06	16.63	15.7	15.3	14.2	8.8	0.0	*P* < 0.0004 (significant)	0.84

**Table 5:** Effect of pH (scale 3 to 11) on the *Lactobacillus helveticus* (BLh) bacteriocin activity against Gram-positive bacteria.

	Gram-positive bacterial strains	Zone of inhibition (mm) vs. pH variations									Pearson's correlation b/w pH variations and activity (*P* value = 0.05 (two-tailed))	*R* squared (*r*^2^)
		3	4	5	6	7	8	9	10	11		
BLh bacteriocin	*E*. *faecium*	10.83	15.80	4.0	3.5	0.9	0.08	0.24	0.04	0.30	*P* = 0.00799 (significant)	0.6589
	*B*. *subtilis*	10.0	15.13	10	5.0	1.2	0.07	0.04	0.04	0.10	*P* = 0.002118 (significant)	0.7621
	*S*. *pyogenes*	9.06	13.83	12.0	10.0	1.5	0.32	0.06	0.02	0.03	*P* = 0.002734 (significant)	0.7451
	*S*. *aureus*	8.23	12.90	25.0	15.0	2.0	0.22	0.29	0.02	0.03	*P* = 0.038482 (significant)	0.4809
	MRSA	6.56	11.96	30.0	20.0	3.56	0.28	0.58	0.42	0.42	*P* = 0.091685 (not significant)	0.3535

**Table 6:** Effect of pH (scale 3 to 11) on the *Lactobacillus helveticus* (BLh) bacteriocin activity against Gram-negative bacteria.

	Gram negative bacterial trains	Zone of inhibition (mm) vs. pH variations									Pearson's correlation (*r*) b/w pH variations and activity (*P* value = 0.05 (two-tailed))	*R* squared (*r*^2^)
		3	4	5	6	7	8	9	10	11		
BLh bacteriocin	*K*. *pneumoniae*	10.66	16.00	8.0	5.0	0.9	0.08	0.24	0.04	0.30	*P* = 0.00287 (significant)	0.7413
	*P*. *aeruginosa*	9.33	15.00	13.0	9.0	1.2	0.07	0.04	0..040	0.01	*P* = 0.003307 (significant)	0.7321
	*A*. *baumannii*	8.66	13.83	17.0	15.0	1.5	0.32	0.06	0.02	0.03	*P* = 0.013958 (significant)	0.6023
	*S*. *paratyphi*	7.33	12.50	22.0	18.0	2.0	0.22	0.29	0.02	0.03	*P* = 0.044299 (significant)	0.462
	*E*. *coli*	5.53	11.6	30.6	25.0	3.56	0.28	0.58	0.42	0.42	*P* = 0.110604 (not significant)	0.3236

**Table 7:** Effect of pH (scale 3 to 11) on the *Lactobacillus plantarum* (BLp) bacteriocin activity against Gram-positive bacteria.

	Gram-positive bacterial strains	Zone of inhibition (mm) vs. pH variations									Pearson's correlation (*r*) b/w pH variations and activity (*P* value = 0.05 (two-tailed))	*R* squared (*r*^2^)
		3	4	5	6	7	8	9	10	11		
BLp bacteriocin	MRSA	10.70	11.00	25.0	20.0	18.0	4.0	3.5	2.5	2.0	*P* = 0.065033 (not significant)	0.4062
	*B*. *subtilis*	10.40	10.33	10.0	10.0	9.0	4.06	4.40	3.56	4.00	*P* = 0.000659 (significant)	0.8281
	*S*. *pyogenes*	10.36	10.13	16.0	14.0	11.0	2.80	2.50	2.0	0.90	*P* = 0.009628 (not significant)	0.6414
	*S*. *aureus*	10.96	11.73	22.0	17.0	16.0	3.5	3.2	3.1	1.5	*P* = 0.033546 (significant)	0.4984
	*E*. *faecium*	12.00	11.26	8.0	8.0	7.0	1.5	1.3	1.2	00	*P* = 0.000028 (significant)	0.9296

**Table 8:** Effect of pH (scale 3 to 11) on the *Lactobacillus plantarum* (BLp) bacteriocin activity against Gram-negative bacteria.

	Gram-negative bacterial strains	Zone of inhibition (mm) vs. pH variations (°C)									Pearson's correlation (*r*) b/w pH variations and activity (*P* value = 0.05 (two-tailed))	*R* squared (*r*^2^)
		3	4	5	6	7	8	9	10	11		
BLp bacteriocin	*K*. *pneumoniae*	10.66	16.00	8.0	5.00	0.9	0.08	0.24	0.04	0.30	*P* = 0.00287 (significant)	0.7413
	*P*. *aeruginosa*	9.33	15.00	13.0	9.00	1.2	0.07	0.04	0.04	0.01	*P* = 0.003307 (significant)	0.7321
	*A*. *baumannii*	8.66	13.83	17.0	15.0	1.5	0.32	0.06	0.02	0.03	*P* = 0.013958 (significant)	0.6023
	*S*. *paratyphi*	7.33	12.50	22.0	18.0	2.0	0.22	0.29	0.02	0.03	*P* = 0.044299 (not significant)	0.4620
	*E*. *coli*	5.53	11.60	30.6	25.0	3.56	0.28	0.58	0.42	0.42	*P* = 0.110604 (not significant)	0.3236

**Table 9:** Effect of bile salts (concentration 0.1 to 0.6) on *Lactobacillus helveticus* (BLh) activity against Gram-positive bacteria.

	Gram-positive bacterial strains	Zone of inhibition (mm) vs. bile salt (solution variations in %)					Pearson's correlation b/w solution variations and activity (*P* value = 0.05 (two-tailed))	*R* squared (*r*^2^)
		0.1%	0.2%	0.3%	0.4%	0.6%		
BLh bacteriocin	*E*. *faecium*	2.20	0.86	0.06	0.07	0.02	*P* = 0.104853 (not significant)	0.6394
	*B*. *subtilis*	2.46	1.06	0.17	0.08	0.03	*P* = 0.089157 (not significant)	0.6729
	*S*. *pyogenes*	2.80	1.36	0.30	0.04	0.07	*P* = 0.081962 (not significant)	0.7012
	*S*. *aureus*	2.96	1.56	0.42	0.09	0.06	*P* = 0.062848 (not significant)	0.7265
	MRSA	3.20	1.90	0.73	0.09	0.06	*P* = 0.043065 (significant)	0.7932

**Table 10:** Effect of bile salts (concentration 0.1 to 0.6) on *Lactobacillus helveticus* (BLh) bacteriocin activity against Gram-negative bacteria.

	Gram-negative bacterial strains	Zone of inhibition (mm) vs. bile salt (solution variations in %)					Pearson's correlation b/w solution variations and activity (*P* value = 0.05 (two-tailed))	*R* squared (*r*^2^)
		0.1%	0.2%	0.3%	0.4%	0.6%		
BLh bacteriocin	*K*. *pneumoniae*	1.73	1.00	0.06	0.06	0.08	*P* = 0.093562 (not significant)	0.6641
	*P*. *aeruginosa*	2.66	1.26	0.08	0.07	0.06	*P* = 0.095045 (not significant)	0.6593
	*A*. *baumannii*	2.83	2.02	0.90	0.08	0.08	*P* = 0.03092 (significant)	0.833
	*S*. *paratyphi*	3.02	2.03	0.47	0.04	0.05	*P* = 0.052663 (not significant)	0.7639
	*E*. *coli*	3.29	2.93	1.06	0.08	0.07	*P* = 0.035733 (significant)	0.816

**Table 11:** Effect of bile salts (concentration 0.1 to 0.6) on *L*. *plantarum* (BLp) bacteriocin activity against Gram-negative bacteria.

	Gram-negative bacterial strains	Zone of inhibition (mm) vs. bile salt (solution variations in %)					Pearson's correlation b/w solution variations and activity (*P* value = 0.05 (two-tailed))	*R* squared (*r*^2^)
		0.1%	0.2%	0.3%	0.4%	0.6%		
BLp bacteriocin	*K*. *pneumoniae*	10.10	8.66	10.30	7.30	6.23	*P* = 0.074956 (not significant)	0.7063
	*P*. *aeruginosa*	15.46	12.76	14.30	7.63	4.23	*P* = 0.020608 (significant)	0.8707
	*E*. *coli*	29.86	10.16	20.66	8.16	7.50	*P* = 0.164037 (not significant)	0.5295
	*A*. *baumannii*	5.43	3.66	4.43	2.80	1.80	*P* = 0.027335 (significant)	0.8446
	*S*. *paratyphi*	25.16	15.50	23.5	7.36	6.36	*P* = 0.092823 (not significant)	0.6646

**Table 12:** Effect of bile salts (concentration 0.1 to 0.6) on *L*. *plantarum* (BLp) bacteriocin activity against Gram-positive bacteria.

	Gram-positive bacterial strains	Zone of inhibition (mm) vs. bile salt (solution variations in %)					Pearson's correlation b/w solution variations and activity (*P* value = 0.05 (two-tailed))	*R* squared (*r*^2^)
		0.1%	0.2%	0.3%	0.4%	0.6%		
BLp bacteriocin	*E*. *faecium*	13.00	9.53	11.80	8.26	6.10	*P* = 0.047788 (significant)	0.7795
	*B*. *subtilis*	18.70	10.23	15.63	7.33	5.23	*P* = 0.081962 (not significant)	0.6897
	*S*. *pyogenes*	22.70	5.23	19.86	4.43	3.33	*P* = 0.229289 (not significant)	0.4314
	MRSA	29.16	0.09	25.8	0.07	0.03	*P* = 0.287817 (not significant)	0.3574
	*S*. *aureus*	25.80	0.90	24.00	1.30	1.06	*P* = 0.302209 (significant)	0.3404

**Table 13:** Effect of UV on *Lactobacillus helveticus* (BLh) bacteriocin against Gram-positive bacteria.

	Gram-positive bacterial strains	Zone of inhibition (mm) vs. time variations (minute)					Pearson's correlation b/w time variations and activity (*P* value = 0.05 (two-tailed))	*R* squared (*r*^2^)
		15 min	30 min	45 min	60 min	75 min		
BLh bacteriocin	*E*. *faecium*	2.9	2.58	2.42	2.37	1.96	0.006 (significant)	0.927
	*B*. *subtilis*	2.0	1.56	1.55	1.32	1.20	0.01453 (significant)	0.8981
	*S*. *pyogenes*	0.9	0.73	0.61	0.42	0.18	0.000424 (significant)	0.99
	*S*. *aureus*	5.0	5.05	4.98	4.98	4.58	0.084817 (not significant)	0.6838
	MRSA	3.97	4.15	4.01	3.78	3.48	0.087703 (not significant)	0.6768

**Table 14:** Effect of UV on *Lactobacillus helveticus* (BLh) bacteriocin against Gram-negative bacteria.

	Gram-negative bacterial strains	Zone of inhibition (mm) vs. time variations (minute)					*P* value from Pearson's correlation b/w time variations and activity (*P* value = 0.05 (two-tailed))	*R* squared (*r*^2^)
		15 min	30 min	45 min	60 min	75 min		
BLh bacteriocin	*K*. *pneumoniae*	6.75	5.42	5.71	5.92	6.62	0.916535 (not significant)	0.0043
	*P*. *aeruginosa*	5.02	4.50	4.27	3.84	5.26	0.94018 (not significant)	0.0022
	*A*. *baumannii*	30.32	10.46	8.23	6.23	18.49	0.451567 (not significant)	0.1997
	*S*. *paratyphi*	5.86	4.41	4.44	4.27	5.48	0.833179 (not significant)	0.0173
	*E*. *coli*	16.02	15.42	15.33	14.66	14.44	0.003904 (significant)	0.9579

**Table 15:** Effect of enzymes activity on a crude extract of bacteriocin.

Enzyme	Crude bacteriocin from *L*. *plantarum* (BLp)	Crude bacteriocin from *L*. *helveticus* (BLh)
Proteinase K	-	-
Pepsin	-	-
Trypsin	-	-
Chymotrypsin	-	-
Papain	-	-
Pronase	-	-
Alpha-amylase	+	+

## Data Availability

The data used to support the study are available from the corresponding author upon request.

## Supplementary Materials

Supplementary Materials Table S1: identification of *Lactobacillus* spp. based on physical and biochemical characteristics and antagonistic activity of bacteriocin extracts against Gram-positive and Gram-negative indicator organisms using the agar well diffusion method. Table S2: antagonistic activity of partially purified bacteriocin against Gram-negative clinical pathogens by agar well diffusion. Table S3: antagonistic activity of partially purified bacteriocin against Gram-positive clinical pathogens by agar well diffusion. Figure S1: effect of bacteriocin against methicillin-resistant *Staphylococcus aureus*. Figure S2: effect of temperature on bacteriocin against methicillin-resistant *Staphylococcus aureus*. Figure S3: effect of temperature on bacteriocin against *E. coli*. Figure S4: effect of pH on bacteriocin against methicillin-resistant *Staphylococcus aureus*. Figure S5: effect of pH on bacteriocin against *Acinetobacter baumannii*. Figure S6: effect of bile salt on bacteriocin against methicillin-resistant *Staphylococcus aureus*. Figure S7: effect of bile salts on bacteriocin against *Acinetobacter baumannii*. Figure S8: effect of UV on bacteriocin against *Acinetobacter baumannii*.
