# Supplementary material for: Characterisation of Bacteriocins Produced by Lactobacillus spp. Isolated from the Traditional Pakistani Yoghurt and Their Antimicrobial Activity against Common Foodborne Pathogens
Source: Biomed Res Int. 2020 Sep 12;2020:8281623. doi: 10.1155/2020/8281623 (PMC7503110; doi:10.1155/2020/8281623)
Supplement: Supplementary Materials — Table S1: identification of Lactobacillus spp. based on physical and biochemical characteristics and antagonistic activity of bacteriocin extracts against Gram-positive and Gram-negative indicator organisms using the agar well diffusion method. Table S2: antagonistic activity of partially purified bacteriocin against Gram-negative clinical pathogens by agar well diffusion. Table S3: antagonistic activity of partially purified bacteriocin against Gram-positive clinical pathogens by agar well diffusion. Figure S1: effect of bacteriocin against methicillin-resistant Staphylococcus aureus. Figure S2: effect of temperature on bacteriocin against methicillin-resistant Staphylococcus aureus. Figure S3: effect of temperature on bacteriocin against E. coli. Figure S4: effect of pH on bacteriocin against methicillin-resistant Staphylococcus aureus. Figure S5: effect of pH on bacteriocin against Acinetobacter baumannii. Figure S6: effect of bile salt on bacteriocin against methicillin-resistant Staphylococcus aureus. Figure S7: effect of bile salts on bacteriocin against Acinetobacter baumannii. Figure S8: effect of UV on bacteriocin against Acinetobacter baumannii. [file 8281623.f1.pdf]

Supplementary

Table S1. Identification of *Lactobacillus spp.* based on physical and biochemical characteristics and antagonistic activity of bacteriocin extracts against Gram-positive and negative indicator organisms using the agar well diffusion method.

Biochemical and Physiological characteristics	*Lactobacillus helveticus*	*Lactobacillus plantarum*
Colony colour	White	Yellow
Cell shape	Rod in short chains	Rods
Gram type	Positive	Positive
Spore type	Negative	Negative
Capsule type	Negative	Negative
Motility test	Positive	Positive
Pigmentation	Negative	Positive
Indole test	Negative	Negative
Methyl red test	Negative	Negative
Citrate utilization test	Positive	Positive
Hydrogen sulphide test	Negative	Negative
Nitrate reduction test	Negative	Negative
Oxidase test	Negative	Negative
Catalase test	Negative	Positive
Nacl Tolerance	Positive	Negative

Table S2 . Antagonistic activity of partially purified bacteriocin against Gram-negative clinical pathogens by agar well diffusion

	**Measurement of zones of inhibition(mm)Standard Deviation**				
**Bacteriocin**	**Clinical isolates of Gram-negative bacteria**				
	Klebsiella pneumonia	*Pseudomonas aeruginosa*	*Acinetobacter baumanni*	*Salmonella paratyphi A*	*E.coli*
**LBp**	1.6±0.3	16.3±0.12	29.35±0.1	16.6±0.5	5.25±1
**LBh**	2.52±0.06	15.3±0.12	24.35±0.1	13.5±1	17.65±0.5

Table S3. Antagonistic activity of partially purified bacteriocin against Gram-positive clinical pathogens by agar well diffusion

	**Measurement of zones of inhibition(mm)Standard Deviation**				
**Bacteriocin**	**Clinical Isolates of Gram-Positive Bacteria**				
	Enterococcus feaciumDO	*Bacillus.subtilis*	*Streptococcus pyogenes*	*Staphylococcus aureus*	*MRSA*
**LBp**	13.6±0.1	10.45±0.1	2.6±0.25	21.2±0.1	13.2±0.6
**LBh**	11.45±1	12.35±0.1	3.52±0.06	19.3±0.15	11.3±0.1

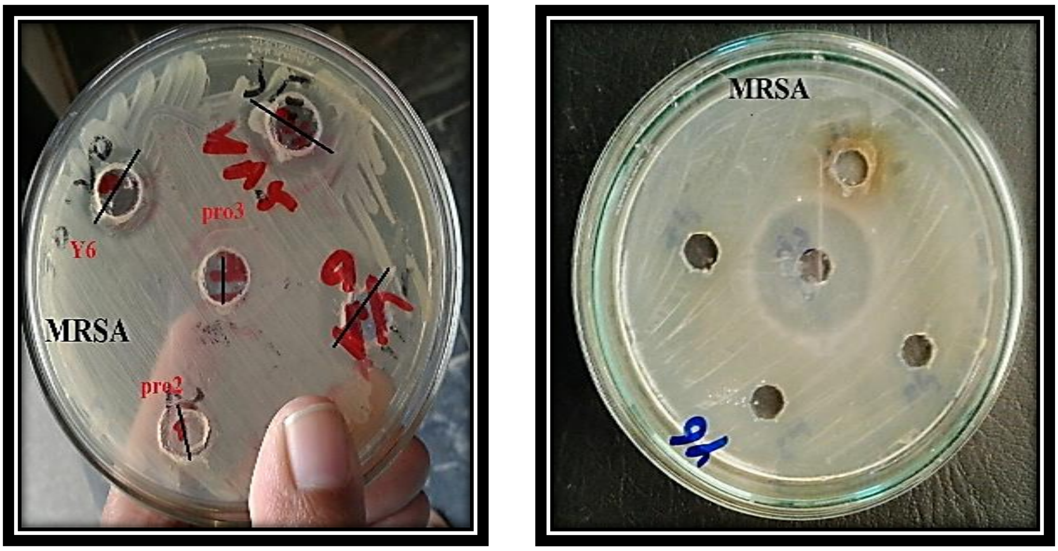

(a) (b)

Figure S 1. Effect of bacteriocin against methicillin-resistant S*taphylococcus aureus*:(a) *Lactobacillus helveticus* (LBh) bacteriocin activity against methicillicn resistance *Staphylococcus aureus ,(*b) *Lactobacillus plantarum* (LBp) bacteriocin activity against methicillicn resistance *Staphylococcus aureus*

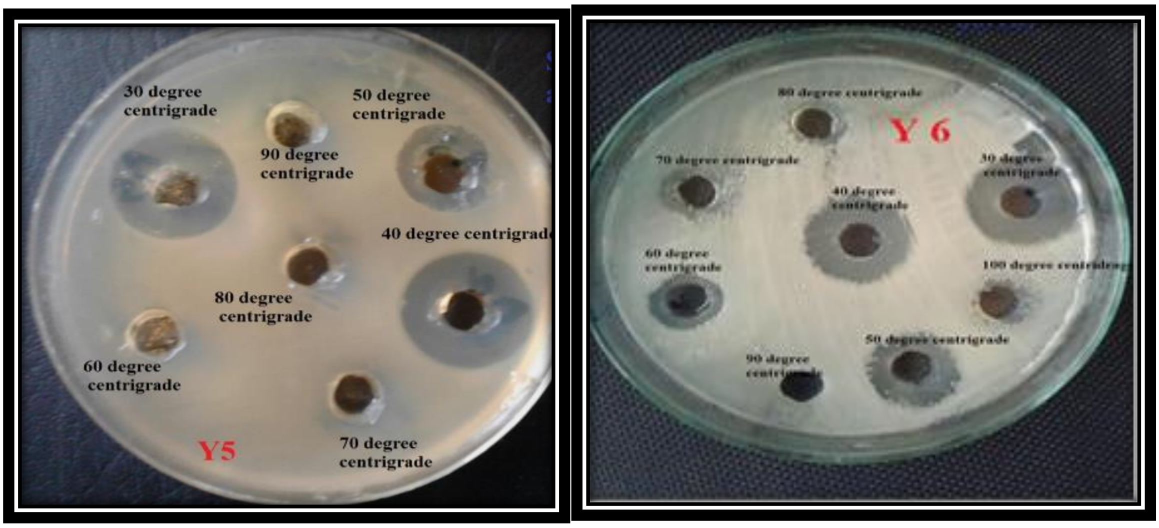

(a) (b)

Figure S2. Effect of temperature on bacteriocin against methicillicn resistance *Staphylococcus aureus* :(a) *Lactobacillus helveticus* (LBh) bacteriocin activity against methicillicn resistance *Staphylococcus aureus ,(*b) *Lactobacillus plantarum* (LBp) bacteriocin activity against methicillicn resistance *Staphylococcus aureus*.

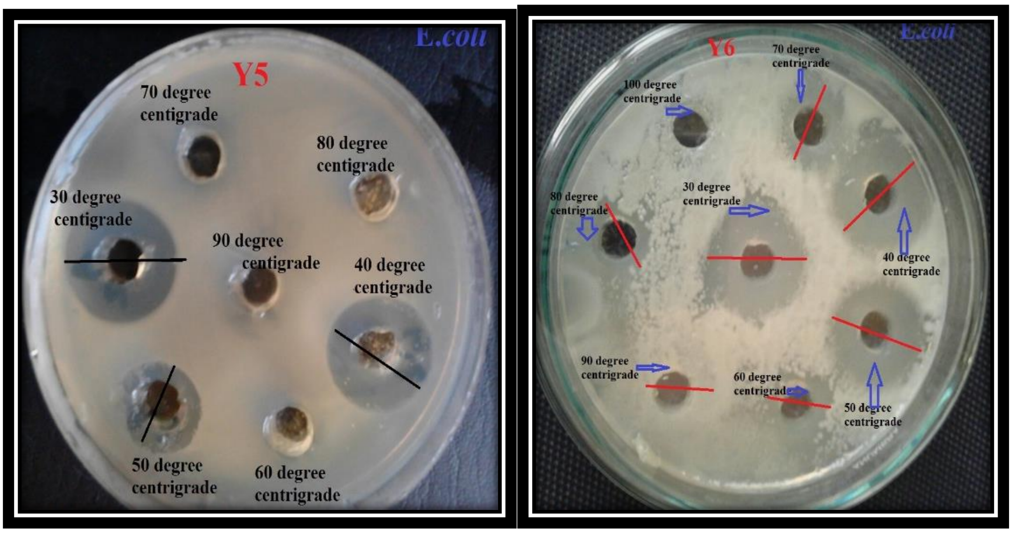

(a) (b)

Figure S3. Effect of temperature on bacteriocin against E.*coli* :(a) *Lactobacillus helveticus* (LBh) bacteriocin activity against E.*coli* ,(b) *Lactobacillus plantarum* (LBp) bacteriocin activity against E.*coli*.

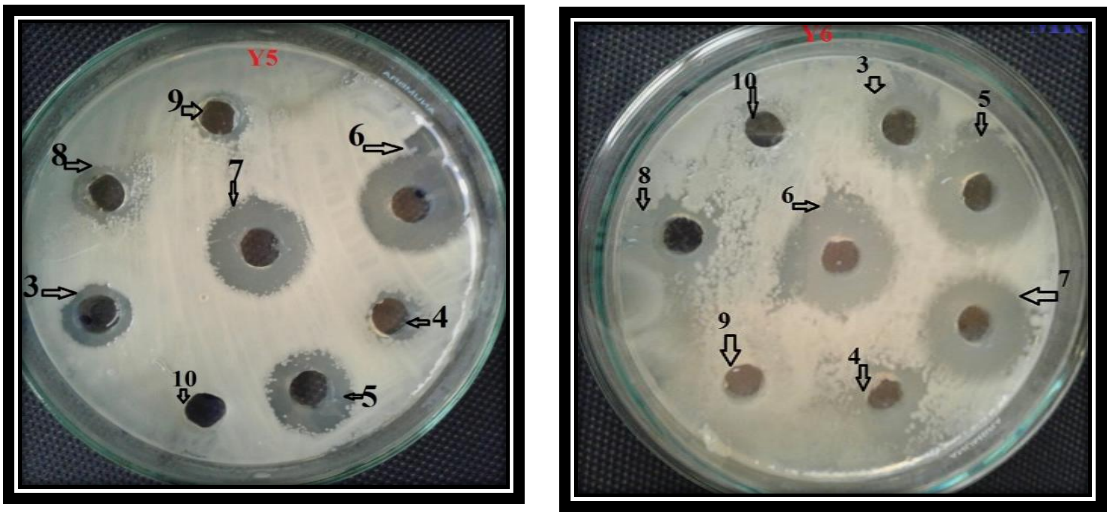

(a) (b)

Figure S4. Effect of pH on bacteriocin against methicillicn resistance *Staphylococcus aureus* :(a) *Lactobacillus helveticus* (LBh) bacteriocin activity against methicillicn resistance *Staphylococcus aureus* ,(b) *Lactobacillus plantarum* (LBp) bacteriocin activity against methicillicn resistance *Staphylococcus aureus*.

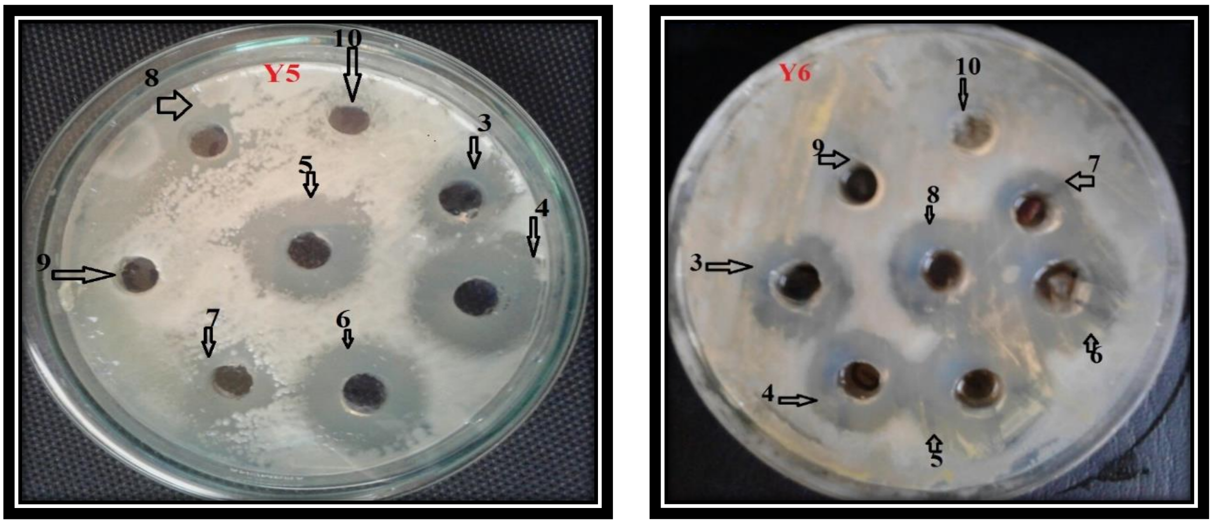

(a) (b)

Figure S5. Effect of pH on bacteriocin against *Acinetobacter*:(a) *Lactobacillus helveticus* (LBh) bacteriocin activity against *Acinetobacter ,(*b) *Lactobacillus plantarum* (LBp) bacteriocin activity against *Acinetobacter*.

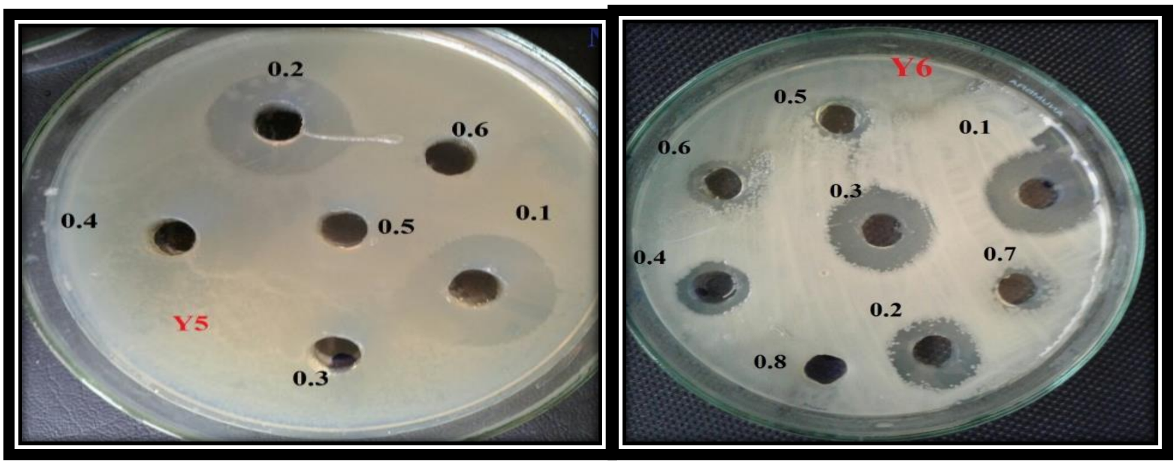

(a) (b)

Figure S6. Effect of Bile salt on bacteriocin against methicillicn resistance *Staphylococcus aureus* :(a) *Lactobacillus helveticus* (LBh) bacteriocin activity against Methicillicn resistance *Staphylococcus aureus ,(*b) *Lactobacillus plantarum* (LBp) bacteriocin activity against Methicillicn resistance *Staphylococcus aureus*.

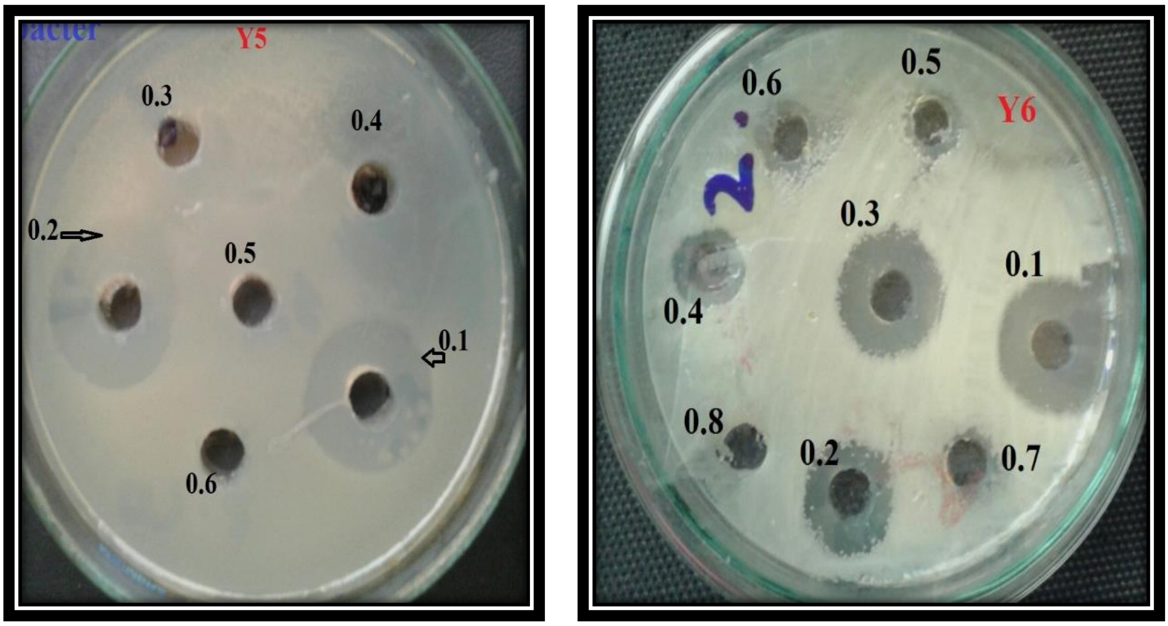

(a) (b)

Figure S7. Effect of bile salts on bacteriocin against *Acinetobacter*:(a) *Lactobacillus helveticus* (LBh) bacteriocin activity against *Acinetobacter* ,(b) *Lactobacillus plantarum* (LBp) bacteriocin activity against *Acinetobacter*.

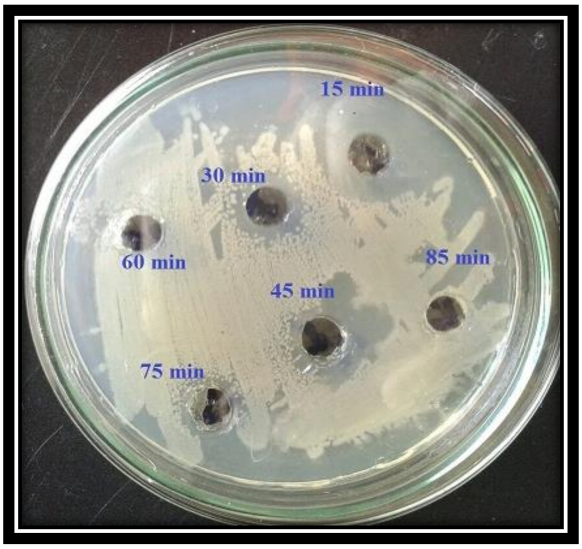

Figure S8. Effect of UV on bacteriocin against *Acinetobacter*:(a) *Lactobacillus helveticus* (LBh) bacteriocin activity against *Acinetobacter* ,
